# Alcohol consumption and atrial fibrillation risk: An updated dose-response meta-analysis of over 10 million participants

**DOI:** 10.3389/fcvm.2022.979982

**Published:** 2022-09-30

**Authors:** Hezi Jiang, Xiaofei Mei, Yufeng Jiang, Jialu Yao, Jinsheng Shen, Tan Chen, Yafeng Zhou

**Affiliations:** Department of Cardiology, Dushu Lake Hospital Affiliated to Soochow University, Medical Center of Soochow University, Suzhou Dushu Lake Hospital, Suzhou, China

**Keywords:** alcohol, atrial fibrillation, meta-analysis, gender difference, risk

## Abstract

**Background:**

The treatment of atrial fibrillation (AF) has made significant progress, but the prevention of AF has not received the attention it deserves. A few recent large-sized studies have conducted dose response analysis and reported different conclusions from previous studies on alcohol consumption and AF risk.

**Objectives:**

The aim of this study is to examine the potential non-linear association between alcohol consumption and risk of AF and explore the potential differences of gender.

**Methods:**

In this updated dose-response meta-analysis, PubMed, Embase and Cochrane databases were searched until June 2022. Risk estimates were reported as relative risk (RR) with 95% confidence intervals (CIs). The random-effects restricted cubic spline models are used to evaluate the potential non-linear association between alcohol consumption and AF risk.

**Results:**

A total of 10,151,366 participants with 214,365 cases of AF enrolled in 13 prospective studies. The overall meta-analysis showed that a 1 drink/day increase in alcohol consumption increased the risk of AF by 6% (RR: 1.06; 95% CI: 1.03–1.08). In gender subgroup analysis, pooled results were different between men (RR: 1.08; 95% CI: 1.05–1.11) and women (RR: 1.05; 95% CI: 0.96–1.14). A linear relationship between alcohol consumption and risk of AF was found in men (*p* = 0.87) while a J-shaped curve was observed in women (*p* = 0.00). Regional subgroup analysis yielded broadly comparable results in Americas (RR: 1.07; 95% CI: 1.03–1.12), Europe (RR: 1.04; 95% CI: 0.99–1.1) and Asia (RR: 1.07; 95% CI: 0.99–1.14).

**Conclusion:**

The relationship between AF risk and alcohol consumption is linear in men, while a potential non-linear J-shaped relationship is shown in women.

**Condensed abstract:**

We conducted a dose-response meta-analysis on the relationship between alcohol consumption and risk of atrial fibrillation. We merged the data of over 10 million participants and found gender differences in the pattern of association with AF and alcohol consumption. The relationship between AF risk and alcohol consumption is linear in men, while a potential non-linear J-shaped relationship is shown in women. In summary, this research is vital in furthering our understanding of the role of alcohol consumption in new-onset AF, especially among different genders.

## Introduction

As the most common clinical arrhythmia, Atrial fibrillation (AF) is influencing over 34 million people worldwide with the increase of risk of stroke, heart failure (HF) and dementia (1, 2), resulting in a tremendous public health burden (3). Although catheter ablation has made substantial progress in the treatment of AF, its prevention has not been given adequate attention. To date, only a few significant risk factors for AF have been identified, like age, male sex, cardiac disease, hypertension, diabetes, obesity and smoking (4). Therefore, the modifiable risk factors that may help reduce the risk of AF must be identified.

There have been several publications (5–8) by far identifying that alcohol has an unambiguous association with AF development, however, some uncertainty may exist over the threshold at which risk is increased. Three previous meta-analyses observed a linear dose-response with an increasing level of alcohol consumption, demonstrating a greater risk of incident AF, and no difference was found between men and women (9–11). Specifically, a study that concluded prospective studies described that the risk of new-onset AF increased by 8% for every 12 g of alcohol intake per day (11). However, several recent studies and the latest meta-analysis failed to demonstrate the link between AF and low to moderate alcohol consumption, and no gender differences was found as well (12–15). A cohort study even suggested using a curvilinear association rather than a linear one to describe this relationship (13). Furthermore, one recent study of more than 9 million participants found the preventive effect of mild drinking on AF (16). Therefore, further research with more focus on alcohol consumption is suggested.

This paper performed an updated dose-response meta-analysis of prospective studies to examine the potential non-linear association of alcohol consumption with the risk of AF and explore the potential gender differences.

## Methods

This meta-analysis was conducted based on the guidelines for the Meta-Analysis Of Observational Studies in Epidemiology (MOOSE) (17).

### Search strategy

The literature of electronic databases (PubMed, Embase, Cochrane) was methodically and comprehensively searched for eligible studies up to June 2022. The following search strategies were used to retrieve articles in English: ‘atrial fibrillation’ and ‘alcohol’. Further manual collection of references attached to retrieved articles was performed to identify other pertinent studies.

### Eligible criteria

The selection was based on the following inclusion criteria: (1) prospective design; (2) at least three types of alcohol consumption were reported; (3) numbers of participants and cases in each type of alcohol consumption were reported; (4) the adjusted relative risk (RR) and the corresponding 95% confidence interval (CI) of incident AF in each type of alcohol consumption were reported.

### Study selection and data extraction

After an electronic search of studies, the retrieved articles were selected with analysis of the title, abstract and full texts progressively according to inclusion criteria. The characteristics and data collected from eligible studies included: author, country, year, sex, age, number of cases and participants, follow-up duration, and types of alcohol consumption.

### Quality assessment

Quality assessment criteria were used to evaluate the quality of eligible studies based on the Newcastle-Ottawa scale (NOS) (18). The scale gives a maximum of nine points for each study: two for comparability, three for result assessment of outcomes, and four for selection. Studies that scored ≥7 were defined as high quality.

### Statistical analysis

STATA version 15.0 was used to perform all statistical analyses. The adjusted RR with corresponding 95% CI for the occurrence of AF in each category of alcohol consumption was considered as the effect size. The I^2^ statistic was adopted to evaluate the heterogeneity among eligible studies with 25, 50, and 75% representing low, moderate, and high heterogeneity, respectively (19). In addition, subgroup analysis was also performed by sex to explore gender differences. The influence of each study on the overall pooled results was observed using sensitivity analysis. Publication bias was assessed with funnel plots and Egger’s regression tests (20). Subsequently, a two-stage random-effect dose-response meta-analysis was conducted to assess the dose-response relationship between AF and alcohol consumption (11, 21, 22). With a generalized least-squares regression method, an alcohol consumption model was established to restrict cubic splines with three knots (23). The null hypothesis that the coefficient of the second spline is equal to zero was tested for calculating a *p*-value of non-linearity. The exposure value refers to the midpoint of the corresponding range of alcohol consumption. The lower boundary was set to zero when the lowest type was open. The midpoint was assumed as 1.5 times the upper boundary if the upper boundary for the highest category was not reported. If one drink corresponded to 12 grams of alcohol, alcohol consumption was converted into drinks/day (11).

## Results

### Literature search

**FIGURE 1:**
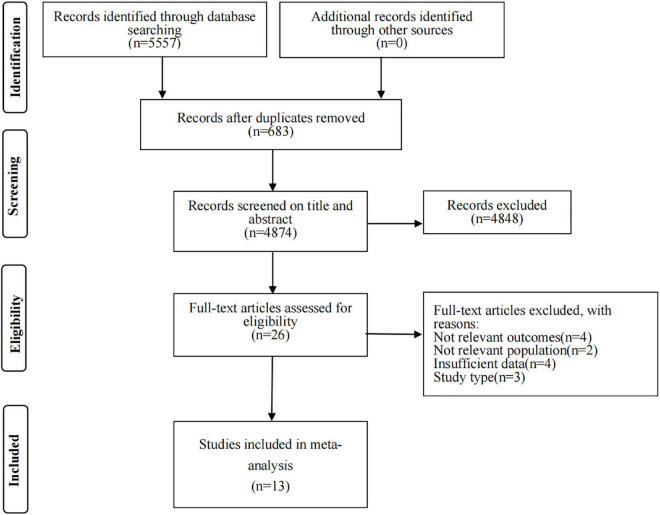
The flow chart of literature identification.

Figure 1 displays a flow chart of the literature search. In short, the initial search strategy retrieved a total of 5,557 citations. After the removal of 683 duplicates, the title and abstract were screened to exclude 4,874 studies. The full-text articles of the remaining 26 citations were screened. Eventually, 13 articles meeting all inclusion criteria were included in the meta-analysis (11–13, 15, 16, 24–31).

### Study characteristics

Table 1 shows the characteristics of eligible studies. Overall, 13 prospective articles were included in the final meta-analysis, representing a total of 10,151,366 participants with 214,365 cases of AF. Both men and women were recruited in the majority of studies except one that recruited women only (27). Table 2 displays the key data of eight studies (11, 12, 24, 25, 27–30) included in the sex-subgroup analysis.

**TABLE 1:** Characteristics and data of included studies.

Author	Country, year	Female (%)	Age range or mean age (years)	Number of cases	Number of participants	Follow-up (years)	Methods of collecting alcohol consumption	Categories of alcohol consumption	Adjustments	NOS
Djousse et al. (24)	United States, 2004	49.1	28–62	1,055	4,672	>50	Interview	None 0.1–12.0 g/day 12.1–24.0 g/day 24.1–36.0 g/day > 36.0 g/day	Age, systolic BP, history of myocardial infarction, congestive HF, left ventricular hypertrophy, valvular heart disease, education	9
Frost and Vestergaard (25).	Denmark, 2004	53	56	556	47,949	5.8	Questionnaire	1.1 g/day 4.6 g/day 9.4 g/day 15.6 g/day 38.8 g/day	Age, body height, BMI, smoking, systolic BP, treatment for hypertension, total serum cholesterol, education	8
Mukamal et al. (26)	United States, 2007	NA	73.1	1,107	4,502	9.1	Questionnaire	None <1 drinks/week 1–6 drinks/week 7–13 drinks/week ≥14 drinks/week	Age, sex, race, income, height, waist circumference, physical activity, psychoactive medication, diabetes, hypertension, coronary heart disease, congestive HF, total cholesterol level	9
Conen et al. (27)	United States, 2008	100	53.5	653	34,715	12.4	Questionnaire	None <1 drinks/day 1–2 drinks/day ≥ 2 drinks/day	Age, systolic BP, history of hypertension, BMI, smoking, history of diabetes, history of hypercholesterolemia, randomized treatment assignment, exercise, race/ethnicity, education	9
Shen et al. (28)	United States, 2011	43.9	62	296	9,640	4	Questionnaire	None 2 g/day 7 g/day 29 g/day	Age, sex, BMI, systolic BP, hypertension treatment, electrocardiogram, PR interval, significant heart murmur, HF	8
Liang et al. (29)	40 countries worldwide, 2012	29.8	66.4	2,093	30,433	4.7	Questionnaire	<1 drinks/week Men: 1–21 drinks/week; women: 1–14 drinks/week Men: >21 drinks/week; women: > 14 drinks/week	Age, sex, education, geographic region, treatment allocation in the trials, BMI, physical activity, smoking, stress, statin use, medical history of coronary artery disease, stroke or transient ischemic attack, hypertension, diabetes, chronic renal disease, sleep apnea	9
Larsson et al. (11)	Sweden, 2014	NA	64	6,019	68,848	12	Questionnaire	< 1 drinks/week 1–6 drinks/week 7–14 drinks/week 15–21 drinks/week >21 drinks/week	Age, sex, education, smoking, BMI, family history of myocardial infarction, history of coronary heart disease or heart failure, history of diabetes, history of hypertension	9
Sano et al. (30)	Japan, 2014	65.4	57.1	286	6,953	6.4	Interview	None <23 g/day 23–46 g/day 46–69 g/day > 69 g/day	Age, sex, cigarette smoking status, BMI, hypertension, hyperglycemia, hyperlipidemia, major ST-T abnormality, previous myocardial infarction and HF	8
Gemes et al. (12)	Norway, 2017	53.0	52.3	1,374	40,790	8	Questionnaire	None 0–3 drinks/week 3–7 drinks/week >7 drinks/week	Sex, height, BMI, marital status, socioeconomic position, physical activity, smoking, diabetes	9
Di Castelnuovo et al. (13)	Italy, 2017	NA	55	504	20,433	8.2	Questionnaire	None 1–12 g/day 12.1–24 g/day 24.1–48 g/day >48 g/day	Age, sex, smoking, education, income, physical activity, BMI, total cholesterol, total calorie intake, history of cardiovascular disease, hypertension, diabetes	9
Bazal et al. (15)	Spain, 2019	56.8	55–80	241	6,077	4.4	Questionnaire	None Men: <140 g/day; women: <70 g/day 14.9 g/day	Age, sex, intervention group, smoking, BMI, height, physical activity, sleep apnea, depression, diabetes, diastolic and systolic BP, hypertension, non-atherosclerotic coronary disease, HF	9
Kim et al. (16)	Korea, 2020	45.3	≥20	195,829	9,776,956	NA	Questionnaire	None 0–150 g/week 150–210 g/week ≥210 g/week	Age, sex, smoking, regular physical activity, social income, BMI, hypertension, diabetes, dyslipidemia, heart disease, sleep apnoea	9
Johansson et al. (31)	Sweden,2020	50.7	46.3	4,353	99,398	12.9	Questionnaire	<1 drinks/week 1–1.9 drinks/week 2–2.9 drinks/week 3–3.9 drinks/week 4–4.9 drinks/week 5–5.9 drinks/week 6–6.9 drinks/week ≥7.0 drinks/week	Age, education level, hypertension, smoking, diabetes, physical activity, history of myocardial infarction, cholesterol level, BMI	9

NOS, Newcastle-Ottawa scale; BP, blood pressure; BMI, body mass index; HF, heart failure.

**TABLE 2:** Total data of studies included in sex-subgroup analysis.

Author	Country, year	Categories of alcohol consumption	Female			Male		
				
			Number of cases/participants	OR	Person-years	Number of cases/participants	OR	Person-years
Djousse et al. (24)	United States, 2004	None 0.1–12.0 g/day 12.1–24.0 g/day 24.1–36.0 g/day >36.0 g/day	111/598 288/1,652 62/298 24/122 26/136	1 0.95 1.16 1.13 1.25	29,900 82,600 14,900 6,100 6,800	52/257 205/1,132 100/602 58/346 129/584	1 0.94 0.98 1.06 1.33	12,850 56,600 30,100 17,300 29,200
Frost and Vestergaard. (25)	Denmark, 2004	4.1 g/day 12.1 g/day 20.0 g/day 36.1 g/day 68.7 g/day	36/5,084 35/5,064 39/5,099 37/5,090 35/5,084	1 1.09 1.27 1.23 1.14	30,010 29,318 29,363 29,253 29,188	61/4,505 63/4,506 86/4,506 75/4,508 89/4,503	1 1.04 1.44 1.25 1.46	25,696 25,537 25,430 25,581 25,757
Conen et al. (27)	United States, 2008	None <1 drinks/day 1–2 drinks/day ≥2 drinks/day	294/15,370 284/15,758 35/2,228 40/1,359	1 0.95 0.98 1.49	190,588 195,399.2 27,627.2 16,851.6	– – – –	– – – –	– – – –
Liang et al. (29)	40 countries worldwide, 2012	<1 drinks/week F: 1–14 drinks/week F: > 14 drinks/week M: 1–21 drinks/week M: > 21 drinks/week;	418/7,464 112/1,546 5/54 – –	1 1.23 1.77 – –	35,080.8 7,266.2 253.8 – –	761/11,311 – – 759/9,593 38/465	1 – – 1.12 1.28	53,161.7 – – 45,087.1 2,185.5
Sano et al. (30)	Japan, 2014	None <23 g/day 23–46 g/day 46–69 g/day >69 g/day	164/3,949 17/530 2/53 0/10 1/4	1 0.9 1.21 0 3.56	25,633 3,322 320 69 35	18/524 19/614 26/625 22/470 16/174	1 0.91 1.22 1.47 3.14	3,289 3,830 3,884 2,939 1,088
Gémes et al. (12)	Norway, 2017	None 0–3 drinks/week 3–7 drinks/week >7 drinks/week	234/3,719 268/14,216 50/3,143 10/536	1 0.98 1.08 1.34	29,651 118,391 25,780 4,304	113/1,583 45,710,576 175/5,248 67/1,769	1 1.1 1.09 1.46	12,043 86,843 43,214 14,130
Kim et al. (16)	Korea, 2020	None 0–150 g/week 150–210 g/week ≥210 g/week	71,554/3,308,938 10,144/988,109 990/96,557 404/40,176	1 0.89 0.93 0.91	27,162,218 8,178,539 798,447 331,985	44,569/1,707,780 37,585/2,148,445 16,234/654,406 14,349/632,545	1 0.96 1.05 1.19	13,752,782 17,607,743 6,986,097 5,143,048
Johansson et al. (31)	Sweden, 2020	<1 drinks/week 1–1.9 drinks/week 2–2.9 drinks/week 3–3.9 drinks/week 4–4.9 drinks/week 5–5.9 drinks/week 6–6.9 drinks/week ≥7.0 drinks/week	948/22,000 329/15,127 175/6,712 60/2,588 30/1,589 24/1,098 9/623 15/680	1 0.88 0.93 0.88 0.94 1.01 0.68 0.95	283,800 185,138.3 86,584.8 33,385.2 20,498.1 14,164.2 8,036.7 8,772	802/126,66 333/5,948 357/6,853 336/6,300 291/5,741 185/3,315 136/2,352 323/5,806	1 1.06 1.07 1.11 1.12 1.16 1.28 1.19	163,391.4 76,729.2 88,403.7 81,270 74,058.9 42,763.5 30,340.8 74,897.4

### Alcohol consumption and atrial fibrillation risk

**FIGURE 2:**
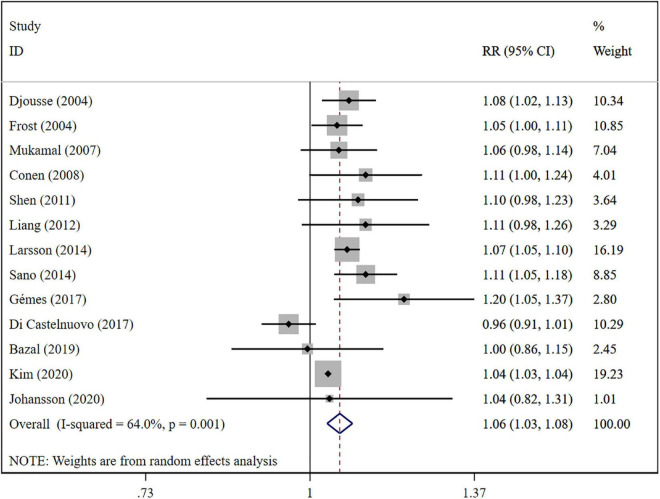
Forest plot of relative risks of atrial fibrillation per 1 drink/day increment in alcohol consumption.

**FIGURE 3:**
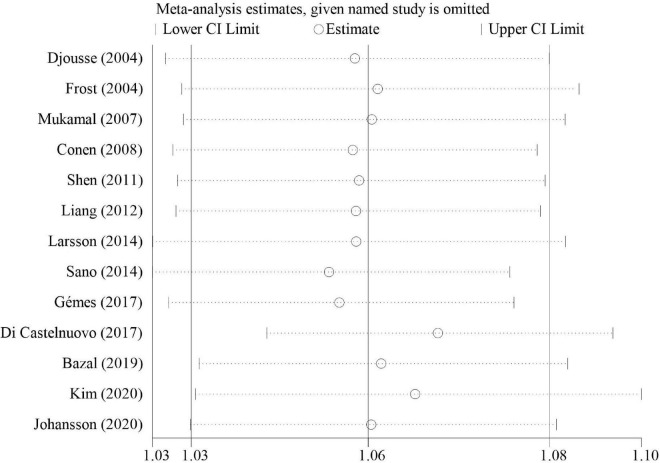
Sensitivity analysis of relative risks of atrial fibrillation per 1 drink/day increment in alcohol consumption.

**FIGURE 4:**
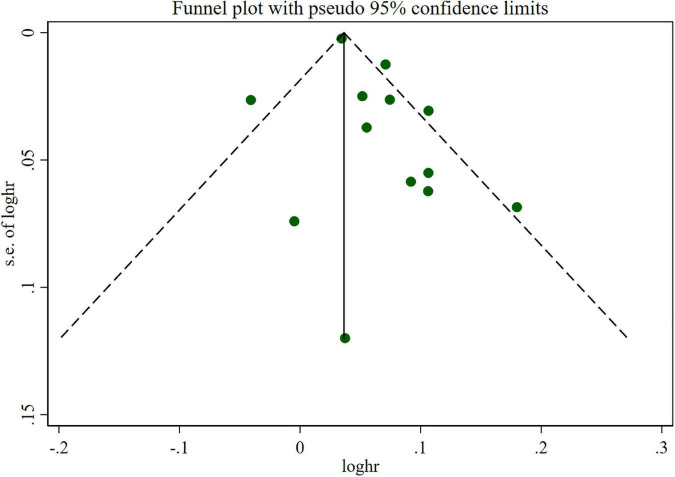
Funnel plots of relative risks of atrial fibrillation per 1 drink/day increment in alcohol consumption.

**FIGURE 5:**
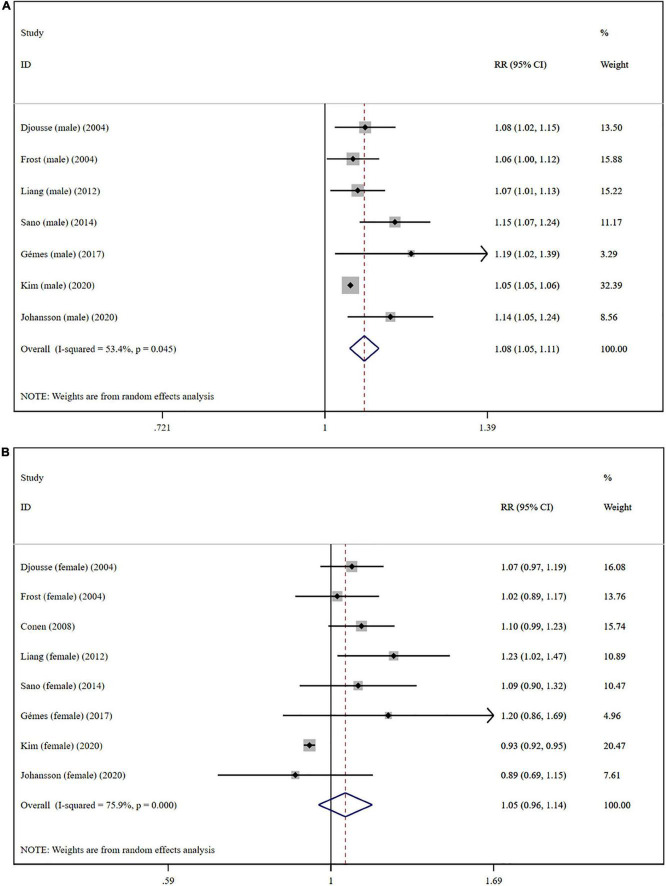
Subgroup analysis of relative risks of atrial fibrillation per 1 drink/day increment in alcohol consumption in different gender. **(A)** Men; **(B)** women.

**FIGURE 6:**
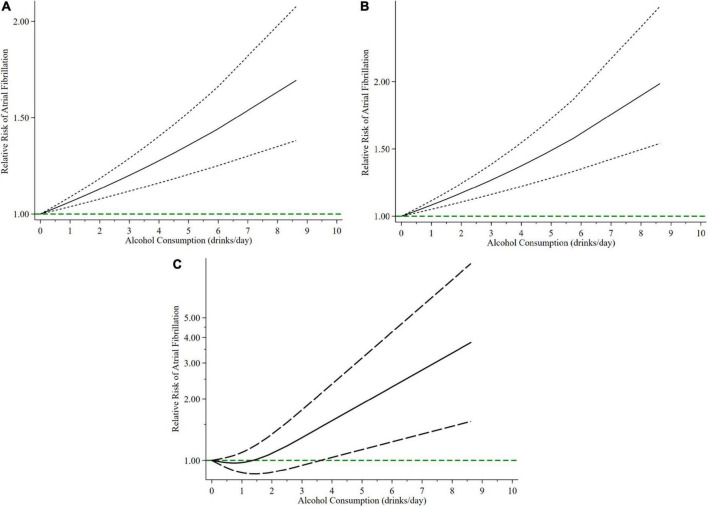
The relationship between relative risks of atrial fibrillation with different alcohol consumption. Continuous line shows non-linear association and long-dashed lines depict 95% confidence intervals. **(A)** In total; **(B)** men; **(C)** women.

**FIGURE 7:**
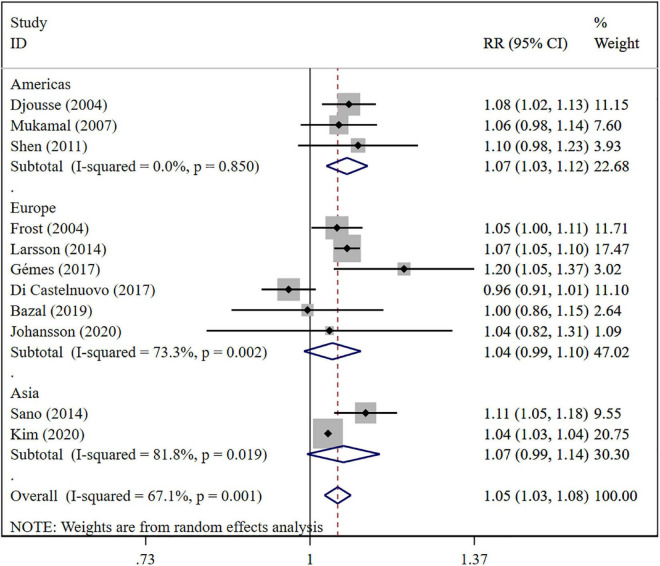
Subgroup analysis of relative risks of atrial fibrillation per 1 drink/day increment in alcohol consumption in different region.

The overall meta-analysis showed that a 1 drink/day increase in alcohol consumption increased the risk of AF by 6% (RR: 1.06; 95% CI: 1.03–1.08) (Figure 2). There was moderate heterogeneity between studies (*I*^2^: 64.0%). Sensitivity analysis (Figure 3) indicated that our results were stable and reliable. Funnel plots (Figure 4) and Egger’s test (*p* = 0.07) suggested no evidence of publication bias. In subgroup analysis (Figures 5A,B), pooled results were different between men (RR: 1.08; 95% CI: 1.05–1.11) and women (RR: 1.05; 95% CI: 0.96–1.14). Moreover, there was also a linear dose-response association between alcohol consumption and risk of incident AF according to a dose-response meta-analysis (*p* = 0.7) (Figure 6A). A similar linear relationship was also observed in men (*p* = 0.87) (Figure 6B). However, a non-linear J-shaped curve was found in women (*p* = 0.00) with a higher risk of AF at alcohol consumption more than 1.4 drinks/day (Figure 6C). Regional subgroup analysis yielded broadly comparable results, Americas (RR: 1.07; 95% CI: 1.03–1.12), Europe (RR: 1.04; 95% CI: 0.99–1.1) and Asia (RR: 1.07; 95% CI: 0.99–1.14) (Figure 7).

## Discussion

This study is an updated dose-response meta-analysis for evaluating the association between AF risk and alcohol consumption. Considering the shortcomings of previous meta-analyses (9–11, 14), the latest comprehensive meta-analysis of high-quality prospective studies and almost 10 million participants was conducted, along with subgroup and sensitivity analysis, to obtain more reliable results. Specifically, five more studies (12, 13, 15, 16, 31) involving a total of 9,943,654 participants with 202,301 cases were included in our study on the basis of the last meta-analysis published in 2014 (11). The newly included studies had lager sample sizes than those before, and two studies conducted dose-response analysis (12, 13).

In total, our results showed a 6% increase in AF risk for per drink one day, which is inconsistent with three previous meta-analyses (9–11), but the risk increased linearly with alcohol consumption remained found. Moreover, subgroup analysis suggested that the pattern of responses to alcohol consumption may vary differently between men and women. One drink/day increase in alcohol consumption is not significantly associated with the development of AF in women. In addition, a linear association was found in men, while a J-shaped curve was observed in women. It can be considered that drinking at any dose may increase the risk of AF in men. For women, however, more than 1.4 drinks/day may increase the risk of AF in women, and less than 1.4 drinks/day even may have a protective effect. Our findings confirmed gender difference in pattern of response to alcohol consumption, which are partially consistent with a recent meta-analysis (14). This meta-analysis found that an average of 1–2 standard drinks per day (moderate alcohol intake) had an increased AF risk compared with lower alcohol intake for men, but not for women. A study only for women also discovered this potential non-linear relationship and confirmed that less than 2 alcoholic drinks/day cannot induce an increased risk of AF (27).

Alcohol may contribute as a trigger of AF and long-term regular consumption promotes progressive atrial remodeling (32). Acute alcohol consumption, as a trigger, may act on electrophysiological milieu for AF through cellular and autonomic effects, such as shortening atrial and pulmonary vein action potential (33, 34), shortening atrial effective refractory period (35), and slowing atrial conduction (35). In addition, habitual drinking induces AF by directly affecting the left atrial substrate and interacting with other risk factors (left ventricular dysfunction, obstructive sleep apnea, hypertension, etc.) (32).

The protective effect of drinking (less than 1.4 drinks/day) in women needs further validation. There may be a variety of reasons for the observed gender difference in this study. Firstly, different dose-response associations may be related to different types of alcoholic beverage consumption. Ethanol and polyphenols in wine may be synergistic, thereby reducing the risk of chronic cardiovascular disease (CVD) (36). It was also found that the proportion of women drinking wine was significantly higher than that of men (31). Secondly, a recent study found that drinking frequency could be an important risk factor for new-onset AF, with an even greater influence than the amount of alcohol consumption in each drinking session (16). Indeed, men tend to have more frequent drinking habits. Thirdly, most of the included studies collected information about alcohol consumption through questionnaires or interviews, which may different social implications for men and women. In response to questionnaires, participants tend to underestimate their actual alcohol consumption (37). Fourth, AF is less prevalent in middle-aged women than in men (38), which may affect the results to a certain extent.

As for the regional subgroup, there was not enough data for a dose-response analysis. Some researches (28, 39, 40) found that dietary exposures other than alcohol, for example caffeine, fish, omega-3 polyunsaturated fatty acids, do associate with risk of AF recent years. As known, there are obvious differences in dietary structure among the United States, Asia and Europe, so some other dietary factors like caffeine which were not be adjusted might influence the subgroup analysis result.

The sample size of more than 10 million is the advantage of meta-analysis in this study, providing high statistical capacity to detect the relationship between AF risk and alcohol consumption. Moreover, all eligible studies are designed prospectively to minimize the likelihood of selection bias and recall bias. Nevertheless, there are also some limitations in this meta-analysis that may affect conclusions. First, although no statistical evidence of publication bias was detected, the likelihood of publication bias cannot be excluded due to the small number of studies. Second, there was moderate heterogeneity in preliminary meta-analysis, which needs to be considered when interpreting the results. The lack of corresponding exposure data prevented us from addressing the moderate heterogeneity. Third, due to the complex mechanism of AF, it is difficult to guarantee that our results are not affected by other confounding factors.

In summary, this study provides further information about the role of alcohol consumption in new-onset AF, particularly between different genders. Regrettably, it is difficult to give a recommended amount of alcohol intake to prevent AF based on the current data.

## Conclusion

The relationship between AF risk and alcohol consumption is linear in men, while a potential non-linear J-shaped relationship is shown in women. An individual participant data meta-analysis of harmonized data across individual studies could help address some of the uncertainties in the shape of the relationship between different types of alcohol consumption and AF risk.

## Data availability statement

The original contributions presented in this study are included in the article/supplementary material, further inquiries can be directed to the corresponding authors.

## Author contributions

HJ, XM, and YJ contributed equally to the study. XM conceived of the presented idea. HJ interpreted the data being published and made figures. HJ and YJ wrote the manuscript with support from XM, JY, and JS. TC and YZ revised the manuscript. All authors contributed to the article and approved the submitted version.

## Abbreviations

AF: atrial fibrillation
HF: heart failure
MOOSE: Meta-Analysis Of Observational Studies in Epidemiology
RR: relative risk
CI: confidence interval
NOS: Newcastle-Ottawa scale
CVD: cardiovascular disease.
